# Interventional radiology practice in a tertiary hospital in South Africa: A 5-year record review

**DOI:** 10.4102/sajr.v29i1.3151

**Published:** 2025-06-30

**Authors:** Gaone Madingwane, Dale K. Creamer

**Affiliations:** 1 Department of Radiation Medicine, Faculty of Health Sciences, University of Cape Town, Cape Town, South Africa

**Keywords:** interventional radiology, interventional procedures, image-guided interventions, utilisation, public hospital, South Africa

## Abstract

**Background:**

Interventional radiology (IR) is a rapidly advancing speciality which uses various imaging modalities to guide diagnostic and therapeutic procedures. Because of its many proven benefits, IR is becoming an integral part of clinical medicine, often taking preference over traditional therapies. While a vast amount of data have been published on IR experiences, there is a paucity of data from South Africa. An objective understanding of the scope and trajectory of our local IR practice is warranted given the global increasing demand for IR services.

**Objectives:**

This study investigated the scope of IR practice in a tertiary public hospital in South Africa over a period of 5 years.

**Method:**

Records for all IR procedures performed at Groote Schuur Hospital (GSH) from 01 January 2019 to 30 December 2023 were retrieved from the hospital Picture Archiving and Communications System (PACS) and radiographers’ records.

**Results:**

The unit performed a total of 7438 procedures ranging from body, biliary, urology, angiography to oncology interventions over the 5-year period. Across all years, abscess drainages were consistently the most common procedure, followed by percutaneous transhepatic cholangiogram (PTC) drainages and nephrostomies.

**Conclusion:**

The unit plays a major role in infection control with an increase in oncologic interventions in line with international practices. There is room for growth in refining the role of IR in trauma and other lifesaving emergency procedures.

**Contribution:**

This study provides information on the scope and utilisation patterns of IR services in South Africa and can serve as a baseline for future monitoring and comparison studies.

## Introduction

Interventional radiology (IR) is a globally recognised and growing speciality that uses various imaging modalities to guide diagnostic and therapeutic procedures.^1^ Generally, the procedures are performed under local anaesthesia or conscious sedation and are held to be cost-effective with low morbidity and mortality rates, offering safer alternatives to many medical and surgical forms of therapy.^2,3^ Types of procedures performed vary among institutions depending on the availability of trained personnel and equipment, but the most common, according to a recent global survey, include biopsies, percutaneous abscess drainages, angiography and vascular access.^4^

Interventional radiology has many uses in various clinical settings, including the management of acute conditions and emergencies, such as treating various causes of haemorrhage through arterial embolisation, relieving nephro-ureteric obstruction, managing acute cholangitis and biliary obstruction and draining abscesses to treat sepsis.^5,6,7^ In the non-emergency setting, IR can be used for the placement of indwelling vascular catheters and ports for long-term vascular access, reproductive interventions such as uterine fibroid embolisation and treating varicoceles, treatment of venous thromboembolism through catheter-guided thrombolysis and inferior vena cava (IVC) filter placement and treatment of various hepatobiliary and urological conditions.^5,8^ With advances in IR techniques and imaging modalities, IR now plays an integral role in the clinical multidisciplinary care team managing oncology patients. The role spans from acquisition of tissue samples through image-guided percutaneous biopsy to local tumour therapy through procedures such as trans-arterial chemo-embolisation (TACE), trans-arterial radioembolisation (TARE) and radiofrequency ablation (RFA), as well as pain management.^9,10,11^

In select patients, many clinicians consider IR as the treatment of choice over traditional surgical procedures because of its minimally invasive nature and proven improved outcomes, specifically, less post-procedural complications, faster recovery times and reduced hospital stay.^12,13^ Other studies have demonstrated the overall value of IR in delivering cost-effective solutions making it a valuable practice in resource-limited settings.^14^ In one study, White investigated the difference in costs between IR procedures and traditional surgical procedures, concluding that IR services cost as low as half of that of traditional surgical therapy for the same procedures. The remarkable differences are because of the different settings under which the procedures are performed and the amount of human resources required.^13^

Despite the many proven benefits of IR, more than half of the world’s population still has no access to IR services, while in sub-Saharan Africa, over 1 billion people have limited or no access to IR.^15^ Because of the lack of data on the availability of IR services in most low- to middle-income countries, it is difficult to measure and objectively comment on the current overall state of IR practice, access and utilisation in these places.^16^ In contrast, access to IR is expected in many developed health systems.^16^ There are many international publications demonstrating upward utilisation trends with throughput doubling every 2–4 years in some countries as well as an increasing number of IR radiologists.^3,9,10^ North America has the largest IR service, while the Asia-Pacific is the fastest growing.^17^ Studies indicate that there are more radiologists in a single academic centre in the United States than there are for the entire population of many low- to middle-income countries.^18^

South Africa currently has approximately 73 interventional radiologists (as of October 2023), serving a population of approximately 62 million (as of the 2022 census).^19,20^ The discrepancies between local IR practice and that of developed countries are multi-factorial, ranging from a lack of infrastructure, high costs of acquiring and maintaining IR equipment, a lack of interventional radiologists and shortage of training programmes.^21^ While several private and public hospitals in South Africa offer IR services, there are limited publications on the types of procedures, techniques used, procedural successes, patient outcomes and utilisation trends. This is true for many low- and middle-income countries. The authors are aware of two recently published studies performed at two academic hospitals which sought to determine local diagnostic reference levels for common fluoroscopically guided IR procedures. Although not the primary objective, these two studies conducted at Chris Hani Baragwanath Academic Hospital in the Gauteng province (the largest hospital in Africa) and at Tygerberg Hospital (a teaching hospital affiliated with Stellenbosch University in the Western Cape province) reflected on the types and frequency of IR procedures performed at these institutions.^22,23^

With the apparent global increase in demand for IR services, there is a need to document the scope of local IR practice and monitor its evolution and trajectory over time with new developments in our health system as well as with international advancements in medicine. Furthermore, the data will provide the basis to advocate for industry investment, government support and establishment of local IR training programmes. Driven by the paucity of data in this area, the objective of the current investigation is to uncover data on the status of IR practice in South Africa by assessing the utilisation patterns of IR service in a single public tertiary hospital over a 5-year period. This study should aid in providing an accurate, competitive value proposition for IR in a resource-constrained environment.

## Research methods and design

### Study design and setting

This was a retrospective, single-centre, cross-sectional record review. At the time of writing, the Groote Schuur Hospital (GSH) IR unit was operating in two IR suites. The older suite uses a Siemens Artis Zee Bi Plane floor-mounted unit installed in 2014, and the other a Siemens Artis Zee Mono Plane ceiling-mounted unit installed in 2018.

A wide range of procedures are performed by the IR team and other surgical departments, namely, neurosurgery, vascular surgery, hepatobiliary surgery and others such as the pain clinic. The unit provides both day-time and after hour (24/7) services.

The unit staff consist of one Canadian fellowship-trained interventional radiologist who joined the department in 2023, four radiographers, five nurses and one operations manager. The unit offers training to two rotating radiology registrars at a time, under the University of Cape Town, Master of Medicine in Diagnostic Radiology programme. Depending on the stage of training, the registrars perform various procedures both independently and under the supervision of the interventional radiologist or another senior registrar.

Referrals to the department are through the ‘Vula’ mobile application (a patient referral mobile phone application) and through the hospital ordering portal. Prioritisation and waiting time are on a case-by-case basis. In general, the radiology department has a prioritisation system of classifying cases into P1, P2 and P3 in descending order of emergency.

### Data collection

The data set included all IR procedures conducted only by the radiology department from 01 January 2019 to 31 December 2023. Interventional procedures performed by other departments were excluded. Data were retrieved from the IR unit’s register which includes a record of all the procedures performed in the unit daily.

To corroborate the register, a search was made on the hospital Picture Archiving and Communications System (PACS) to retrieve all reports of IR procedures performed during the specified period. The data were tabulated into a spreadsheet, divided into months and years. Each procedure has a specific code on the physician booking portal, and they are categorised systematically into biliary, urology, angiograms and general. For ease of interpretation, the procedure names, codes and categories were maintained and are presented as they appeared on the booking portal and PACS.

The retrieved data were then filtered to highlight which of the procedures were emergencies (annotated by a radiology registrar as P1 or P2) and which were elective procedures (annotated as P3). Another filter was applied to divide the data set into outpatients and inpatients. The outpatient data were further divided to indicate the various facilities patients were referred from.

For comparison, similar studies from other tertiary hospitals (2 local and 2 international) were reviewed, and the average number of procedures performed at these facilities per year as well as the most frequently performed procedures were tabulated along with those of GSH.

### Data analysis

The frequency of each procedure per year and per category was tabulated on Microsoft Excel. The most frequent procedures were identified and expressed as a percentage of the total for the respective categories. The average number of procedures per year was calculated. The data were further analysed using SPSS version 28. To determine any statistically significant correlations between the frequency of procedures performed across the years, a cut-off value of *p* < 0.05 was used.

### Ethical considerations

Ethical approval to conduct this study was obtained from the Human Research Ethics Committee, Faculty of Health Sciences of the University of Cape Town (reference number: 173/2024). Permission to conduct the research was obtained from Groote Schuur Hospital. Because of the retrospective nature of this study, no patient outcomes were affected.

## Results

### Type and frequency of interventional radiology procedures performed at Groote Schuur hospital

Over the 5-year period, a total of 7438 interventional procedures were performed by the GSH radiology department. The average number of procedures performed per year was 1488.

**FIGURE 1:**
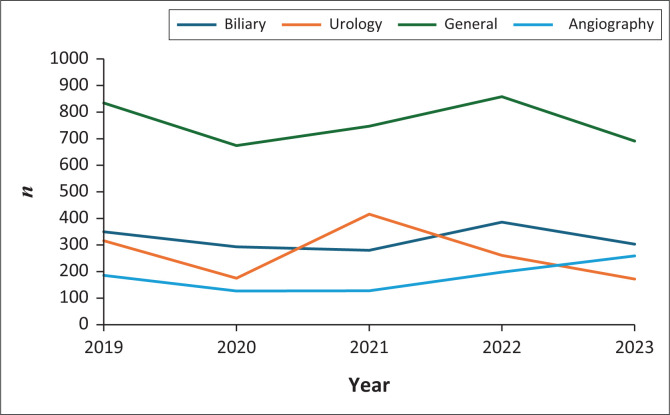
Comparison of the frequency of the four interventional procedure categories performed by the radiology department at Groote Schuur Hospital from 2019 to 2023.

The procedures are categorised into biliary, urology, angiography and general (Table 1) according to the targeted body systems. Across all five years, general category procedures were most frequent, accounting for 51% (*n* = 3799) of all procedures (Figure 1). More specifically, abscess drainage, percutaneous transhepatic cholangiography (PTC) and nephrostomy were the most common procedures over the 5 years accounting for 27% (*n* = 1999), 14% (*n* = 1070) and 9% (*n* = 650), respectively, of the total workload.

**TABLE 1:** Type and frequency of interventional procedures performed by Groote Schuur Hospital radiology department from 2019 to 2023.

Procedure type	2019	2020	2021	2022	2023	Total
**Biliary**						
Spyglass	0	0	0	0	1	1
Stone removal	3	2	1	2	2	10
External drain	9	4	9	8	6	36
Biliary PTA	9	4	5	8	17	43
Internal drain	21	12	9	11	21	74
Internal-external drain	38	42	30	44	35	189
Biliary stent placement	43	36	26	46	34	185
PTC	226	192	200	267	185	1070
**Urology**						
DJ retrieval	0	0	0	0	1	1
Guidewire placement	0	0	6	0	4	10
Nephrostomy tube replacement	24	30	20	8	5	87
Nephrostomy tube removal	44	23	32	24	41	164
DJ stent	62	25	30	32	74	223
Nephrostomy	186	97	123	108	136	650
**Angiography**						
Gastric varices embolisation	0	1	0	0	0	1
Adrenal vein sampling	0	0	0	1	0	1
Ovarian	2	0	0	0	0	2
Thrombin injection for aneurysm	0	1	0	0	1	2
Venogram	1	0	0	2	0	3
Cerebral	3	0	0	0	0	3
BRTO or CARTO	0	0	0	0	3	3
Portal vein embolisation	1	1	3	2	2	9
Aortogram	0	0	0	0	8	8
Neck	2	3	1	3	7	16
Abdomino-pelvic	7	4	4	2	6	23
Prostate artery embolisation	0	6	2	12	8	28
Pulmonary artery embolisation	4	8	3	14	9	38
Renal	7	12	20	10	16	65
TACE	9	11	7	24	30	81
Bronchial artery embolisation	22	20	15	17	13	87
Uterine artery embolisation	55	10	8	8	29	110
Hepatic and/or coeliac	28	19	21	53	73	194
Peripheral angio-embolisation	43	31	44	50	54	222
**General**						
PleurX removal	0	0	0	0	1	1
Radiofrequency ablation	0	0	0	1	0	1
Drain checks	0	0	0	0	1	1
IVC filter insertion	0	0	0	0	1	1
Port removal	0	0	0	0	2	2
Venogram	0	0	0	0	2	2
Port check	0	0	0	3	0	3
Microwave ablation	0	0	1	1	2	4
Port insertion	0	0	0	0	6	6
Bone biopsy	5	0	0	0	2	7
Hickman catheter	0	0	0	0	8	8
PleurX Abdomen	0	0	0	0	10	10
Transjugular liver biopsy	2	0	1	0	9	12
PleurX Chest	0	0	0	0	12	12
Lumbar puncture	0	0	8	4	7	19
Myelogram	7	6	4	6	0	23
Nerve blocks	24	2	1	0	0	27
Chest drain	57	41	88	96	192	474
Aspiration	46	20	21	43	39	169
Drain upsize or replacement	53	36	42	48	37	216
Plugged liver biopsy	94	70	70	41	41	316
Core biopsy	80	98	110	187	11	486
Abscess drainage	465	397	401	428	308	1999
**Total**	1682	1264	1366	1614	1512	7438

PTA, percutaneous transhepatic angiography; PTC, percutaneous transhepatic cholangiogram; DJ, double J; BRTO, balloon-occluded retrograde transvenous obliteration; CARTO, coil-assisted retrograde transvenous obliteration; TACE, trans-arterial chemo-embolisation; IVC, inferior vena cava.

**FIGURE 2:**
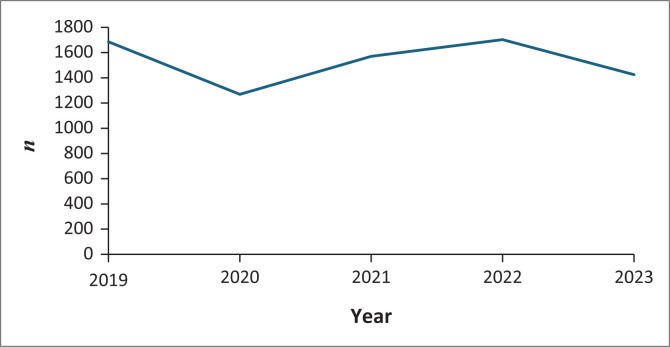
Frequency of interventional procedures performed per year at Groote Schuur Hospital radiology department from 2019 to 2023.

There was a decrease in the number of procedures between 2019 and 2020 (*p* = 0.0018) owing to the coronavirus disease 2019 (COVID-19) pandemic. From 2020 to 2021, the number of procedures increased significantly (*p* = 0.00625), stabilising between 2021 and 2022 (*p* = 0.2246) before dropping again from 2022 to 2023 (*p* = 0.001108) during a transition within the unit when the radiologist who had been operating the unit pursued a fellowship (Figure 2).

### Prioritisation of procedures

**FIGURE 3:**
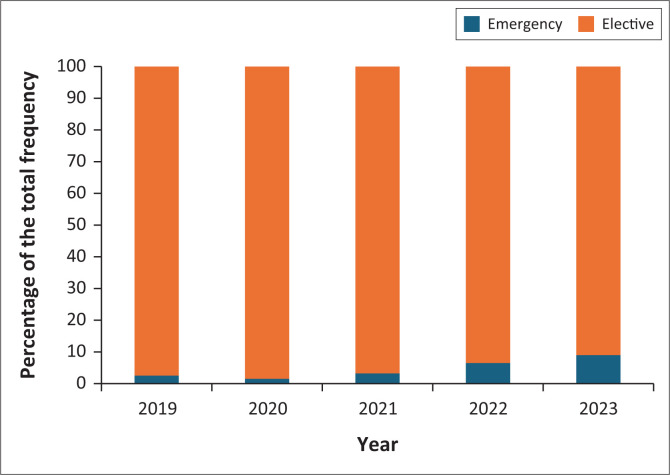
Comparison of priority status of interventional procedures performed by Groote Schuur Hospital radiology department from 2019 to 2023.

Across the years, elective procedures far outweighed emergency procedures. However, elective procedures decreased over the years from 97% of the total procedures in 2019 to 91% of the total procedures in 2023 (*p* < 0.001; Figure 3), indicating a statistically significant increase in emergency procedures over the years.

### Utilisation patterns of Groote Schuur Hospital interventional radiology service

**FIGURE 4:**
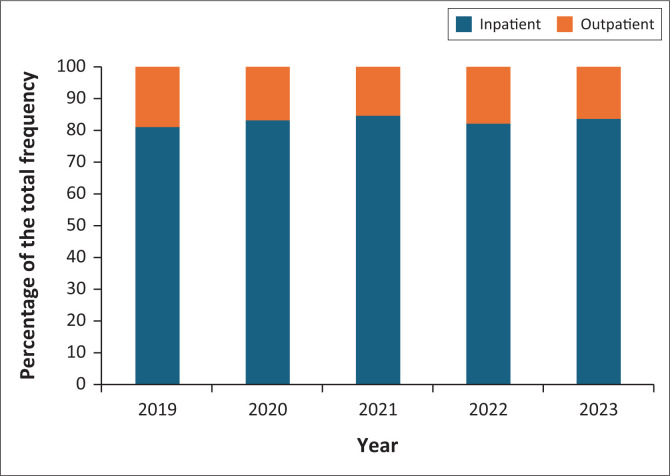
Comparison of frequency of inpatient to outpatient referrals to Groote Schuur hospital interventional radiology unit from 2019 to 2023.

From 2019 to 2023, inpatient referrals far outweighed outpatient referrals (Figure 4). The proportion of outpatient to inpatient referrals did not change significantly over the years (*p* = 0.06).

**FIGURE 5:**
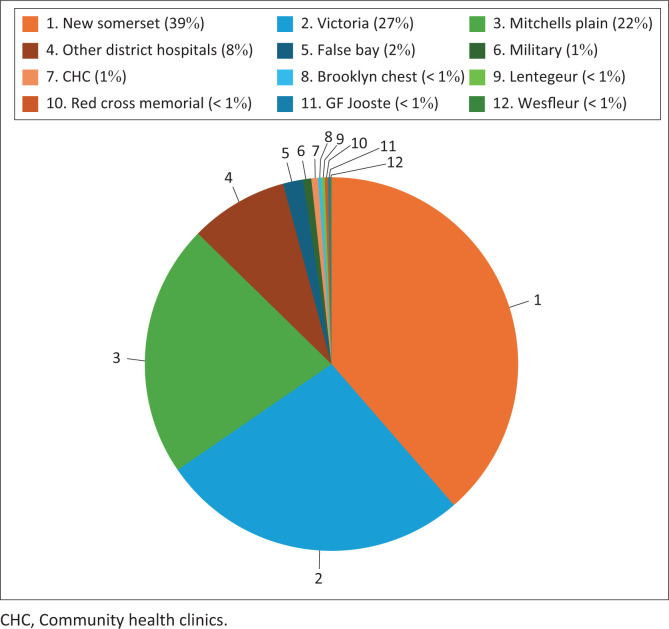
Comparison of proportion of referrals from outside hospitals to Groote Schuur hospital interventional radiology unit from 2019 to 2023.

Across all years, the highest volume of referrals from other facilities were from three referral hospitals: New Somerset accounting for 39% of all external referrals, Victoria and Mitchells Plain hospitals accounting for 27% and 22% respectively (Figure 5).

## Discussion

Interventional radiology is a critical component of any institution delivering high-quality care, providing minimally invasive solutions which have been proven to be safe with low morbidity and mortality rates and lower costs.^5,13,26,27^ The demand for IR service is increasing globally, and many international guidelines and standards of practice highlight the importance of having a well-resourced, 24/7 IR service in hospitals.^8,28^

The output of the GSH IR service compares to that of a 590-bed hospital in Singapore that served a population of more than 700 000 people at the time of the study, compared to GSH, a 975-bed hospital serving a population of approximately 3 million (25). During the 5-year study (2010–2015), the hospital performed a total of 7140 IR procedures with a team of four interventionalists offering a 24/7 IR service. This equates to 4% less than the output at GSH, which currently has only one interventionalist, over the same duration of years though different time frames. Another study conducted in a single cancer centre in New York demonstrated that in 1 year alone, the centre performed 59% more procedures than GSH^24^ (Table 2). This reflects that one of the greatest barriers to IR access in South Africa and perhaps even Africa as a whole, is the lack of qualified personnel.

**TABLE 2:** Comparison of annual number of interventional radiology procedures and most frequent procedure across various tertiary institutions.

Institution	Average number of procedures per annum	Most frequent procedure
GSH, South Africa	1488	Abscess drainage
CHBAH, South Africa^22^	564	PTBD
Tygerberg Hospital, South Africa^23^	815	Aorto-bifemoral angiography
Memorial Sloan Kettering Cancer Center, US^24^	3591	Abscess drainage
Khoo Teck Puat Hospital, Singapore^25^	1428	Drainage procedures

Note: Please see full reference list of this article, Madingwane G, Creamer DK. Interventional radiology practice in a tertiary hospital in South Africa: A 5-year record review. S Afr J Rad. 2025;29(1), a3151. https://doi.org/10.4102/sajr.v29i1.3151, for more information

GSH, Groote Schuur Hospital; CHBAH, Chris Hani Baragwanath Academic Hospital; US, United States; PTBD, percutaneous transhepatic biliary drainage.

The recommendation by the Society of African Interventional Radiology and Endovascular Therapy (SAFIRE) is one interventional radiologist per 1 million population, while South Africa, as of 2023, is at one per 2.7 million,^19^ indicating a huge gap, but also an opportunity to facilitate and motivate the establishment of local training programmes. Inadequate staffing may not only compromise patient safety but also has a negative long-term effect on the physical and mental health of the limited team members having to work excessive hours and provide aid particularly in a centre like GSH which offers a 24/7 IR service. Staff burnout is associated with increased rates of medical errors which in turn result in high expenses to facilities.^28^

Although limited, two recent studies conducted at public, tertiary academic hospitals in South Africa demonstrated the scope of IR practice at these facilities and can be used along with this study to depict the utilisation patterns of IR service in South Africa (Table 2). One study conducted at Chris Hani Baragwanath Academic Hospital (CHBAH) in the Gauteng province (the largest hospital in Africa), demonstrated that in 2019 the hospital performed a total of 564 interventional procedures compared to 1682 performed at GSH in the same year. Unlike at GSH, the most frequent interventional procedure offered at this institution is percutaneous transhepatic biliary drainage accounting for 26% of the workload. Important to note is that during the study period, the CHBAH biplane angiography suite was non-functional for 4 months; hence, these numbers are not a true reflection of the actual hospital IR output.^22^

Another similar study conducted at Tygerberg Hospital in the Western Cape province (affiliated with Stellenbosch University) over a period of 3 years (2015–2018) reported a total of 2446 fluoroscopically guided interventional procedures, averaging 815 per year. The most frequently performed procedures were aorto-bifemoral diagnostic and interventional angiography,^8,23^ which were the least performed category of procedures at GSH. This is because vascular procedures at GSH are shared among IR, vascular surgery and neurosurgery departments, while this study only focused on procedures performed specifically by the IR unit. This is potentially an area of collaboration for registrar and possibly fellowship training in these very specific areas.

Similar to a global survey conducted in 2023^4^ and two other studies conducted in tertiary institutions in the United States and Singapore,^24,25^ percutaneous abscess drainages were the most common procedure performed in the department across all 5 years, demonstrating the significant role that the unit plays in the management of localised infections. The role of IR in infection control, particularly the benefits of percutaneous over operative abscess drainage, has been highlighted in multiple studies indicating lower morbidity and mortality observed with percutaneous drainage, particularly in patients with high anaesthetic risk.^7,29^

The variety of procedures performed has evolved over the years, especially with the addition of a fellowship trained interventional radiologist to the team in 2023. This saw the introduction of procedures such as Spyglass, tunnelled drains (PleurX) and advanced techniques of balloon-occluded retrograde transvenous obliteration (BRTO) and coil-assisted retrograde transvenous obliteration (CARTO). Procedures which were traditionally offered by surgical departments such as transjugular liver biopsy, IVC filter placement and Hickman line placement are also now being performed primarily by the IR unit. Oncology interventions, specifically TACE also demonstrated an overall increase in numbers over the 5 years. This is a major step towards matching international standards of practice where IR now forms part of the multidisciplinary care team caring for cancer patients in addition to surgical, medical and radiation oncologists.^9,10^

There is still room for growth through contributing to the management of acute cases in the emergency setting. With the high trauma rates in Cape Town and the increasing number of emergency cases depicted by the results, the IR unit has a role to play in the non-operative management of splenic, hepatic and renal injuries, as has been proven to be effective in other institutions and international trauma centres.^6^ The results also reflect underutilisation of lifesaving procedures such as emergency uterine artery embolisation (UAE) for the management of post-partum haemorrhage. In reality, the limited availability of personnel makes it difficult to provide a comprehensive IR service including 24/7 coverage for emergency cases.

The Royal College of Radiologists recommends a minimum of 6 radiologists for a population < 1 million to provide 24/7 IR service with provisions for full night cover and an exchange next-day cover to allow for sufficient rest and to avoid patient compromise.^30^ In this regard, GSH is still far from the international standards of practice. At present, the hospital depends on registrars to provide after-hour IR emergency services for cases such as abscess drainages, PTCs and nephrostomies (depending on the registrar’s level of training, skill and comfort with the procedures) with the more complex procedures such as embolisations requiring the expertise of the one interventionalist, who by default is on call daily. Again, the need for more trained personnel is highlighted.

### Study limitations

This study only depicts interventional procedures performed specifically by the IR unit and does not include interventional procedures performed in the ultrasound unit (which include thyroid and lymph node fine needle aspiration and musculoskeletal interventions), CT unit (CT-guided lung and spine biopsies) and mammography unit (breast ultrasound guided biopsies and placement of markers) as well as by other non-radiology departments such as vascular surgery, neurosurgery and hepatobiliary surgery, thus underestimating the actual interventional service at GSH. The studies from other institutions used for comparison had many factors that could account for the variations such as different time periods (the current study included COVID-19 pandemic), staffing and availability of equipment. The introduction of the P1, P2 and P3 prioritisation and annotation system in 2022 also caused discrepancies in the data. Some of the cases were discussed and accepted via ‘Vula’ and had no priority annotation on the PACS, so only the radiographers’ register was used to determine the urgency level of the case. These results may not be a true representation of the ratio of elective to emergency procedures from 2022 onwards.

### Recommendations

For future institutional, national or international comparative studies, there is a need to standardise IR procedure terminology to accurately document the trends in practices. For statistics and future comparison studies, the prioritisation of cases needs to be standardised, and every case must be annotated accordingly. A potential area for future studies is to investigate the time taken between accepting and annotating a case as an emergency to the procedure being done.

## Conclusion

This study depicted the type and frequency of procedures offered by the GSH IR unit and the evolution of the IR service for a period of 5 years. The unit plays a substantial role in infection control and has an increasing contribution to oncologic interventions, which is consistent with international standards of practice. While there has been a progressive increase in the volume and complexity of procedures offered, particularly after the addition of a fellowship trained interventional radiologist in 2023, the unit still has a long way to go in matching the international standards of practice especially in providing acute care and comprehensive, 24/7 service. A significant number of trained interventionalists is still required to ensure the provision of a sustainable, quality standard of care without compromising on patient safety or staff wellbeing.
